# Transcription Factor *GmERF105* Negatively Regulates Salt Stress Tolerance in *Arabidopsis thaliana*

**DOI:** 10.3390/plants12163007

**Published:** 2023-08-21

**Authors:** Lu Li, Zhen Zhu, Juan Liu, Yu Zhang, Yang Lu, Jinming Zhao, Han Xing, Na Guo

**Affiliations:** Key Laboratory of Biology and Genetics and Breeding for Soybean, Ministry of Agriculture, MOE National Innovation Platform for Soybean Bio-Breeding Industry and Education Integration, Zhongshan Biological Breeding Laboratory, State Key Laboratory of Crop Genetics & Germplasm Enhancement and Utilization, College of Agriculture, Nanjing Agricultural University, Nanjing 210095, China; 2019201044@njau.edu.cn (L.L.);

**Keywords:** soybean, transcription factor, *GmERF105*, GCC-box, salt stress, ROS

## Abstract

The Ethylene Response Factor (ERF) transcription factors form a subfamily of the AP2/ERF family that is instrumental in mediating plant responses to diverse abiotic stressors. Herein, we present the isolation and characterization of the *GmERF105* gene from Williams 82 (W82), which is rapidly induced by salt, drought, and abscisic acid (ABA) treatments in soybean. The GmERF105 protein contains an AP2 domain and localizes to the nucleus. GmERF105 was selectively bound to GCC-box by gel migration experiments. Under salt stress, overexpression of GmERF105 in Arabidopsis significantly reduced seed germination rate, fresh weight, and antioxidant enzyme activity; meanwhile, sodium ion content, malonic dialdehyde (MDA) content, and reactive oxygen species (ROS) levels were markedly elevated compared to the wild type. It was further found that the transcription levels of CSD1 and CDS2 of two *SOD* genes were reduced in OE lines. Furthermore, the *GmERF105* transgenic plants displayed suppressed expression of stress response marker genes, including *KIN1*, *LEA14*, *NCED3*, *RD29A*, and *COR15A/B*, under salt treatment. Our findings suggest that GmERF105 can act as a negative regulator in plant salt tolerance pathways by affecting ROS scavenging systems and the transcription of stress response marker genes.

## 1. Introduction

Soil salinization is a significant obstacle to crop growth, as high salt levels negatively impact plant development. Currently, more than 20% of the world’s cultivated land is affected by salt stress, and this figure is on the rise. As soil salinization continues to worsen, salt stress has emerged as a primary abiotic stressor that severely impedes crop growth and development [1,2]. Soybean is a vital food crop used to produce various soy products, including soybean oil, soy sauce, and protein extracts. However, with increasing salt stress, soybean yields are significantly affected [3,4]. The demand for soybeans in China is consistently high each year, and planting soybeans on saline-alkaline soil has emerged as a promising means to increase yields, making the development of salt-tolerant soybean varieties necessary. With the advancement of molecular breeding, genetic engineering is a promising approach to addressing this issue. However, prior to this, it is crucial to understand the mechanisms of plant salt tolerance, which are currently lacking. In order to achieve sustainable development, it is crucial to identify the key components of the plant salt-tolerant network [5].

Salt stress induces an increase in reactive oxygen species (ROS) levels within plant cells by altering ion balance and disrupting metabolic processes in organelles such as mitochondria and chloroplasts, which generate ROS. The resulting ROS accumulation can cause cellular dysfunction and damage to lipids, DNA, and proteins, ultimately leading to growth inhibition and cell death [6,7,8]. Plants have developed sophisticated mechanisms to counteract ROS production and oxidative damage under salt stress, including the activation of antioxidant systems and other pathways. For example, plants increase the activity of antioxidant enzymes, such as superoxide dismutase (SOD), catalase (CAT), and ascorbate peroxidase (APX), to scavenge ROS [9]. In addition, plants regulate gene expression and signaling pathways, such as ion uptake, transport, and allocation, compatible solute synthesis, root growth, and water use, to maintain ion balance and metabolic homeostasis, thereby reducing ROS levels and oxidative damage and protecting the structure of the cell membrane [10,11,12,13]. These regulatory mechanisms enhance the plant’s salt tolerance and reduce the production of malondialdehyde (MDA) and its analogs.

Transcription factors (TFs) are critical regulators of plant growth and development, as well as their response to abiotic and biotic stresses [14]. By binding to specific DNA elements, TFs can directly or indirectly modulate the expression of downstream target genes [15]. Ethylene response factors (ERFs) represent a large family of TFs that can bind to the DRE/GCC elements in the promoter regions of target genes [16]. ERFs have been implicated in various abiotic stress responses, including salt, drought, cold, and heat. For instance, in soybean, the ERF gene *GmERF113* activates the expression of GmPR10-1, a gene involved in drought tolerance, by binding to the GCC-box in its promoter region [17]. Similarly, overexpression of *GmERF3*, which binds to both GCC box and DRE/CRT elements, enhances salinity and dehydration tolerance by upregulating the expression of stress-related genes [18]. The poplar ERF gene, *PalERF109*, is rapidly induced by salt, and overexpression of *PalERF109* enhances salt tolerance by directly activating the expression of the high-affinity K^+^ transporter (HKT) gene [19]. On the other hand, overexpression of *ERF76* in poplar enhances salt tolerance by upregulating the expression of stress-related genes and enhancing the biosynthesis of ABA and GA [20]. In tomatoes, *JERF3* is induced by multiple stresses and acts as a positive regulator of stress-related genes by binding to cis-acting elements associated with osmotic responses and oxidative stress [21]. However, some ERFs can also act as negative regulators of stress responses. For instance, overexpression of *OsERF922* and *OsERF106* in rice reduces salt tolerance by disrupting ion homeostasis and modulating stress-responsive gene expression [22,23]. Conversely, silencing the *StERF3* gene in potatoes enhances the plant’s tolerance to NaCl stress, while overexpressing *StERF3* increases sensitivity to salt, suggesting that *StERF3* is a negative regulator of stress response in potatoes [24]. Furthermore, LlERF110 binds to both GCC and CGG elements, and its overexpression in plants results in delayed bolting and thickening phenotypes, decreased heat resistance, and disrupted ROS homeostasis [25]. It should be noted that the functions of ERF family genes are context-dependent and may vary depending on the specific plant species, type of stress, and growth conditions. Therefore, further research is necessary to fully comprehend the roles of ERF family genes in regulating plant tolerance to abiotic stresses.

In this study, we identified a transcription factor, *GmERF105* (*Glyma.20G070000*), with upregulated expression using an unpublished soybean salt-tolerant RNA-seq dataset. It is hypothesized that the transcription factor *GmERF105* may play a crucial role in the response of plants to salt stress. To assess its involvement in salt stress response, we produced transgenic Arabidopsis plants that heterogeneously overexpressed *GmERF105*. The role of *GmERF105* in response to salt stress was elucidated by analyzing the performance of these plants under salt stress.

## 2. Results

### 2.1. Isolation and Sequence Analysis of the GmERF105 Gene

**Figure 1:**
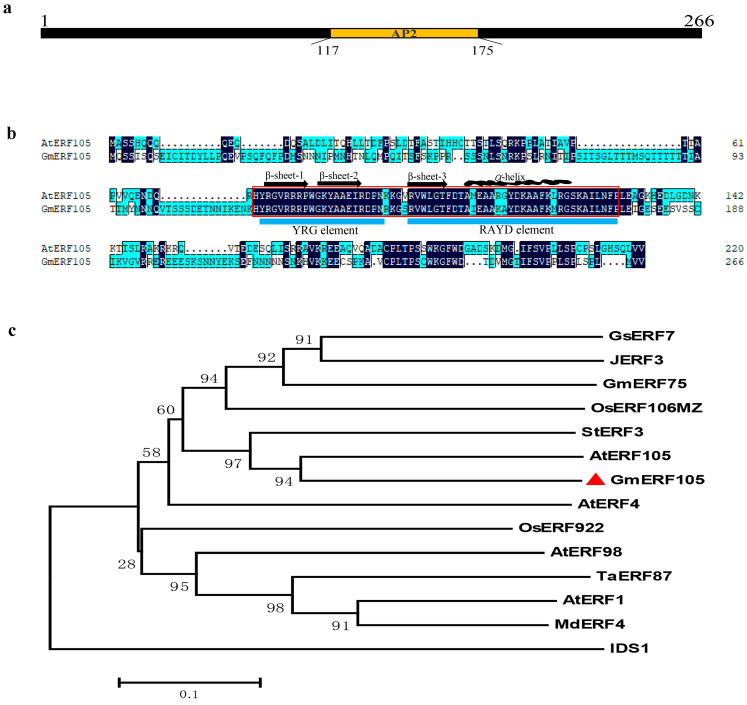
GmERF105 protein sequence and phylogenetic tree analysis. (**a**) Protein structure of the GmERF105; (**b**) The sequence alignments generated by DNAMAN software. The red box indicates the AP2 DNA-binding domain. The amphipathic α-helix and three β-sheets are labeled above the corresponding sequence. The YRG and RAYD elements are represented by the blue bar below the alignment; (**c**) Phylogenetic relationships between GmERF105 and other ERFs from various species. The phylogenetic tree was generated by the neighbor-joining method using MEGA 6.0. GmERF105 is indicated by the red triangles. The protein sequences of the selected ERF genes were obtained from Phytozome or Genebank; the Accession Numbers are as follows: GsERF7 (GLYMA_07G044300), JERF3 (AAQ91334.1), GmERF75 (GLYMA_10G016500), OsERF106MZ (OSNPB_080537900), StERF3 (XP_006365342.1), AtERF105 (AT5G51190), GmERF105 (GLYMA_20G070000), AtERF4 (AT3G15210), OsERF922 (Os01g0752500), AtERF98 (AT3G23230), TaERF87 (XP_044360011.1), AtERF1 (AT3G23240), and MdERF4 (XP_008364034.2), IDS1 (OSNPB_030818800).

The full-length cDNA sequence of *GmERF105* was isolated from Williams 82. Sequence analysis showed that *GmERF105* contained an 801-bp open reading frame encoding a polypeptide of 266 aa with a predicted molecular weight of 29.83 kDa and an isoelectric point of 9.03. The GmERF105 protein is predicted to contain a typical AP2 domain, ranging from 117 to 175 amino acids (Figure 1a). Compared with ERF proteins in Arabidopsis thaliana, the AP2 domain is highly conserved. Further structural analysis revealed that the AP2 domain contains two conserved fragments, YRG and RAYD, forming three β-folds and one α-helix, respectively, and the former can interact with base pairs in the DNA helix (Figure 1b). Sequence alignment and phylogenetic tree analysis showed that *GmERF105* has the highest similarity with *AtERF105*, a member of the B-3 subgroup in *Arabidopsis thaliana*, so it was named *GmERF105*. In addition, *GmERF105* is in a branch with *StERF3* from potatoes (Figure 1c). Since *StERF3* has previously been reported to negatively regulate salt tolerance in plants, we hypothesize that *GmERF105* may also play a role in plants exposed to salt stress.

### 2.2. The Expression Patterns of GmERF105 in Soybean

**Figure 2:**
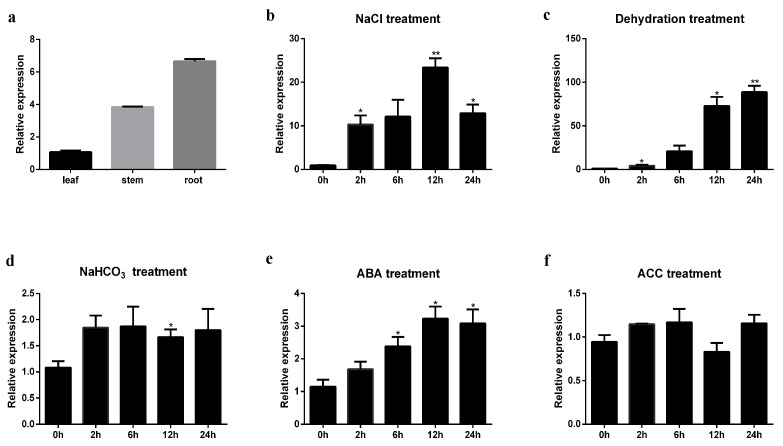
Expression of *GmERF105* in soybean tissues under different abiotic stress treatments. (**a**) the expression levels of *GmERF105* in different soybean tissues were detected. When the first trifoliate leaves are fully unfolded, the roots, stems, and leaves are selected as samples. (**b**–**f**) patterns of *GmERF105* expression under the different treatments of abiotic stresses; (**b**) 150 mM NaCl; (**c**) dehydration; (**d**) 50 mM NaHCO_3_; (**e**) 100 µm ABA; (**f**) 50 µm ACC. Roots were collected at 0, 2, 6, 12, and 24 h after different treatments, respectively. The values are the means ± SDs (*n* = 3). The asterisks show significant differences between the control and salt treatments according to Student’s *t*-test: * *p* < 0.05; ** *p* < 0.01.

Quantitative real-time PCR (qRT-PCR) was performed to assess the transcript levels of *GmERF105* in W82 plants. The results showed that *GmERF105* was expressed in the soybean roots, stems, and leaves (Figure 2a). The expression patterns of *GmERF105* were further analyzed in soybean seedlings during salt (150 mM NaCl), dehydration, NaHCO_3_ (50 mM), and two hormone treatments (100 uM ABA and 50 uM ACC). The abundance of *GmERF105* mRNA was rapidly induced when the seedlings were treated with salt and then gradually increased until reaching a peak at 12 h, an approximately 24-fold increase relative to the initial level (Figure 2b). Dehydration resulted in rapid accumulation of *GmERF105* mRNA levels, which reached their maximum level at 24 h with an approximately 70-fold increase relative to the initial level (Figure 2c). In addition, the expression level of *GmERF105* was slightly induced at 2 h under ABA treatment and gradually increased until reaching a peak at 12 h, with a 3.4-fold increase relative to the initial level (Figure 2e), while there was no significant change under ACC and NaHCO_3_ treatment (Figure 2d,f).

### 2.3. GmERF105 Is a Nuclear Protein

**Figure 3:**
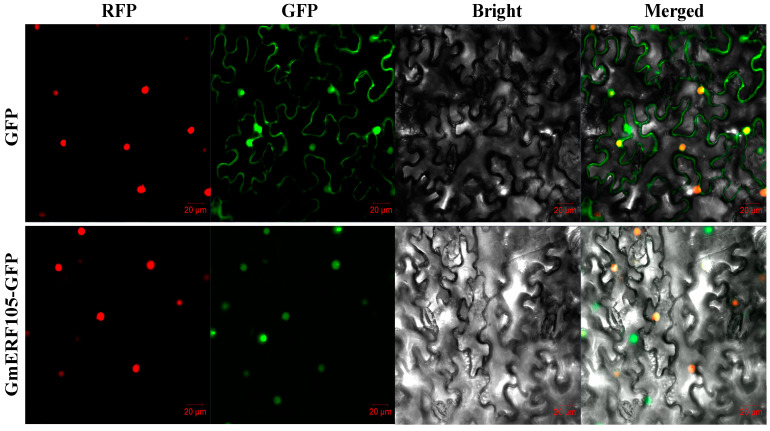
GmERF105 is a nuclear protein. Nuclear localization of the GmERF105 protein in leaf epidermal cells of *Nicotiana benthamiana*. Nicotiana leaves transiently expressing GFP alone (**upper**) and GmERF105-GFP (**bottom**) proteins were observed with a confocal microscope (Olympus FluoView FV1000, Tokyo, Japan).

To determine the subcellular localization of the GmERF105 protein, it was analyzed by transiently expressing a gene encoding a GmERF105-GFP fusion protein under the control of the CaMV35S promoter in tobacco epidermal cells. An empty vector (pBinGFP4) was used as a control. As shown in Figure 3, the green fluorescence signal from the GmERF105-GFP fusion protein was co-localized with the red fluorescence signal from the red fluorescence protein (RFP) fusion nuclear localization gene. While the signal of GFP fluorescence was distributed throughout the whole cell. These results indicated that GmERF105 is localized in the nucleus.

### 2.4. GmERF105 Binds Specifically to the GCC-Box

**Figure 4:**
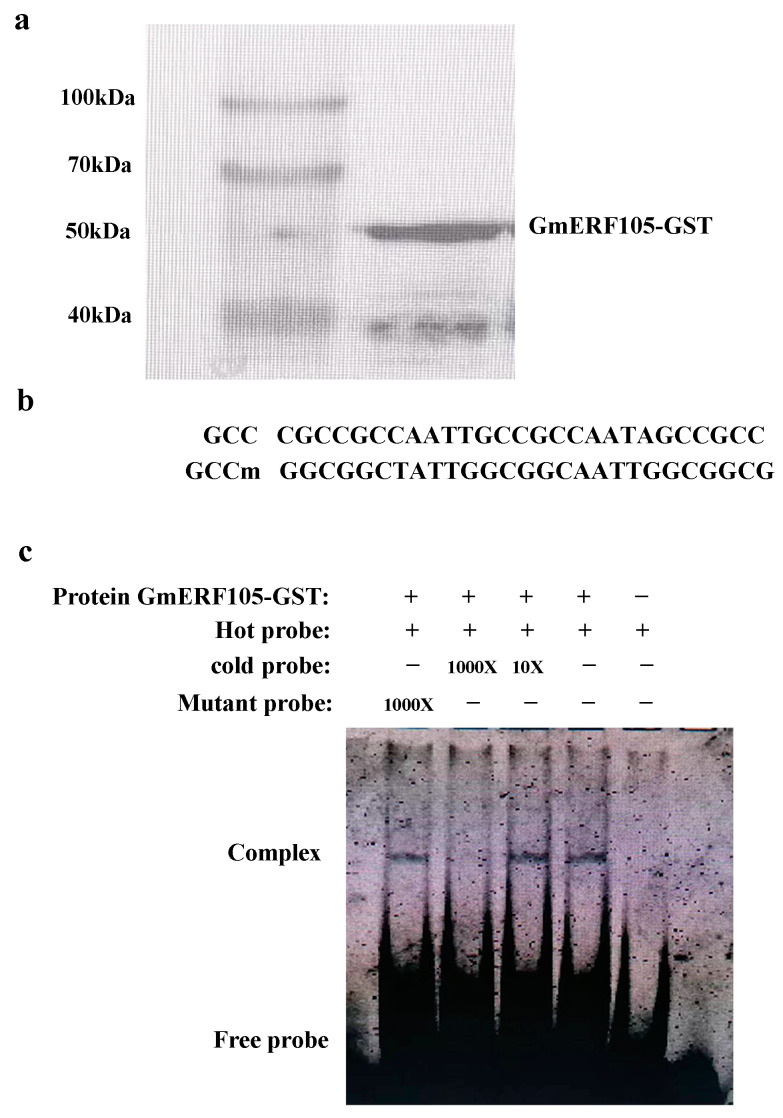
Characterization of the DNA binding affinity of the recombinant GmERF105 protein. (**a**) western about the recombinant GmERF105 protein. (**b**) sequence of the oligonucleotides used in the DNA binding studies. (**c**) gel retardation assay showing sequence-specific binding of the recombinant GmERF105 protein. The black band indicates the position of a protein–DNA complex after the incubation of 6 ‘FAM-labeled DNA probes and the GmERF105 protein. The bottom part is the free probe. Hot probe, a probe labeled with 6 ‘FAM. Cold probe, is the same as the labeled probe, but it is not labeled, as a competitor. Mutant Probe: unlabeled DNA probe containing the mutated motif.

It has been shown that the ERF protein, which contains the ERF/AP2 DNA binding motifs, can bind to the GCC box [26]. To test the binding activity of GmERF105 to this cis-acting element, we synthesized recombinant GmERF105 protein in *Escherichia coli* and fused it with glutathione S-transferase (GST) (Figure 4a). The ability of recombinant GmERF105 fusion proteins to bind cis-acting elements was tested using a gel mobility shift assay. As shown in Figure 4b, GmERF105 is combined with the GCC box. The DNA binding specificity of GmERF105 was also confirmed in competitive experiments (Figure 4b,c). The 1000× unlabeled GCC sequences effectively competed for the specific interaction between GmERF105 and the labeled GCC sequences. In contrast, mutant GCC sequences did not affect the GmERF105 binding activity of GCC sequences.

### 2.5. GmERF105 Represses Seed Germination of Transgenic Plants under Salt Condition

**Figure 5:**
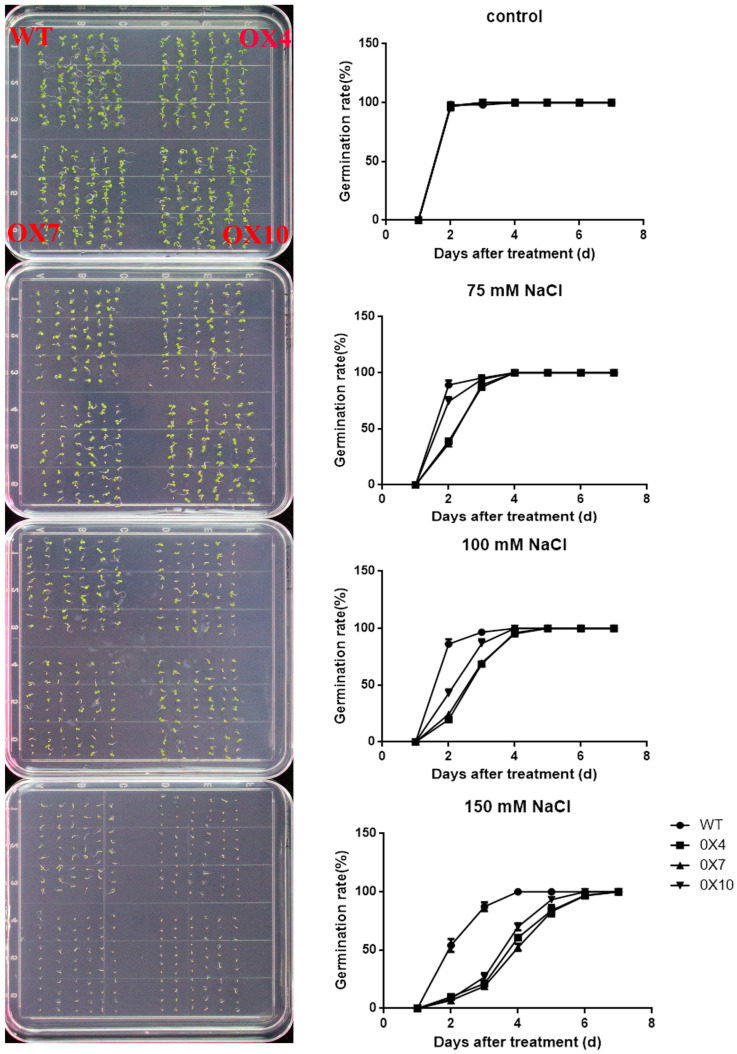
Overexpression of *GmERF105* repressed the germination rates of Arabidopsis under salt stress. Seed germination under the treatment of salt. The photo was taken on the fourth day. Error bars represent ± SD. The observation values were the averages of three repetitions (*n* = 3). Three independent biological experiments were carried out to investigate the seed germination of WT and *GmERF105* transgenic lines under salt stress. WT, wild type; OX4, 7, 10: *GmERF105* Arabidopsis transgenic lines of T3 generations.

To investigate the effect of *GmERF105* under salt stress, we generated transgenic *A. thaliana* overexpressing *GmERF105*. The seeds of the three T3 homozygous lines (OE-2, OE-7, OE-10) were obtained for analysis (Figure S1). To investigate the function of *GmERF105* in transgenic plants at the germination stage under salt conditions, seeds were germinated on MS medium either without NaCl or supplemented with 75 mM, 100 mM, and 150 mM NaCl. After 7 days, the germination rate of each line was recorded, and the seeds of all strains germinated without salt treatment. On the contrary, under salt conditions, *GmERF105*-overexpressing lines showed lower germination rates than WT (Figure 5). Taken together, these results suggested that overexpressed *GmERF105* repressed the germination of transgenic plants under salt stress.

### 2.6. GmERF105 of Transgenic Plants Plays a Negative Role in Combating Salt Tolerance

**Figure 6:**
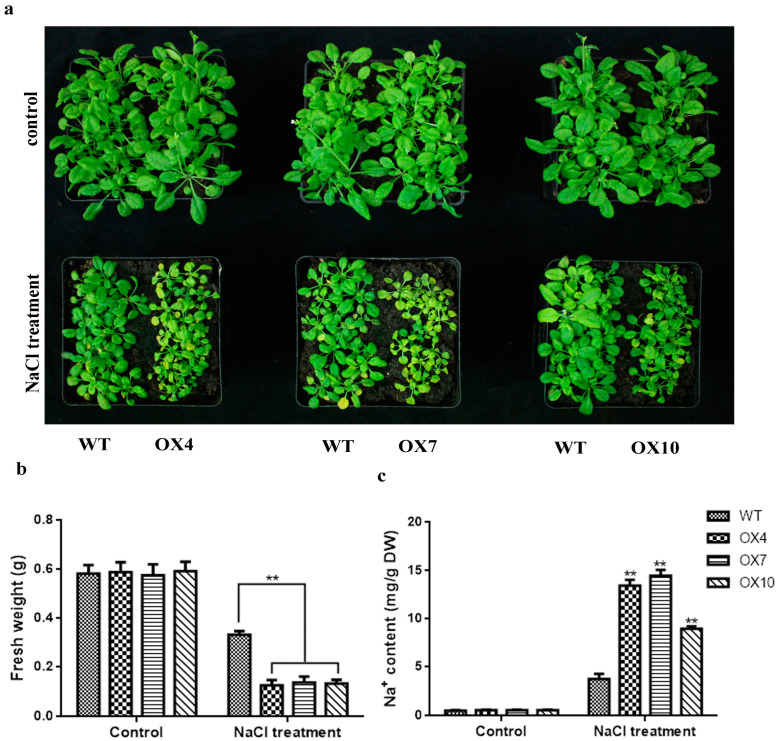
*GmERF105* decreased the resistance of Arabidopsis thaliana to salt stress. (**a**) the phenotypes of WT and *GmERF105* transgenic lines under normal and salt stress. (**b**) the determination of fresh weight (aboveground). (**c**) the determination of Na^+^ contents. The wild-type controls and *GmERF105* transgenic line plants were grown in pots for two weeks and then irrigated with a solution of 150 mM NaCl for 16 days. WT, wild type; OX4, 7, 10: *GmERF105* Arabidopsis transgenic lines of T3 generations. The data are mean values ± SDs (** *p* < 0.01; Student’s *t*-test). All the experiments included three biological replications.

After the plant had six leaves, it was watered using 150 mm NaCl solution. H_2_O was used for the control group. Then the plants were observed to grow until different phenotypes appeared. After 15 days, the plants under normal conditions were growing well. However, under salt treatment, overexpressed lines displayed more sensitivity than WT (Figure 6a). We tested aboveground fresh weight and Na^+^ content without salinity stress, and there was no significant difference between OE plants and WT. The Na^+^ content of the OE plants after the salt stress treatment was significantly greater than that of WT (Figure 6c), and the aboveground fresh weight is also much less than that of WT; the fresh weight of WT decreased by 42.68%, while that of OE plants decreased by 77.26% (Figure 6b). Taken together, these results indicated that overexpression of *GmERF105* decreases salt tolerance in transgenic Arabidopsis.

### 2.7. GmERF105 Represses Antioxidant Capacity under Salt Stress

**Figure 7:**
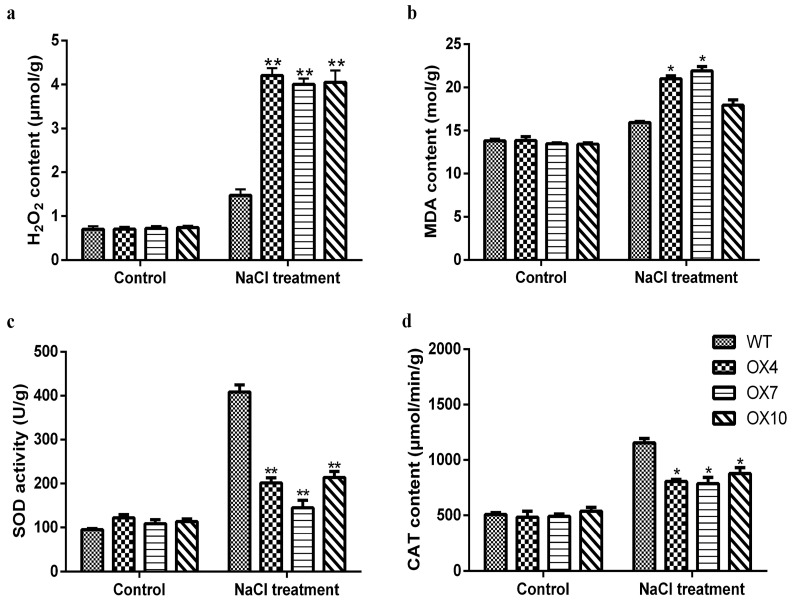
Analysis of H_2_O_2_, MDA, and antioxidant enzyme activity of WT and *GmERF105*-overexpressing plants. (**a**) H_2_O_2_ contents of WT and *GmERF105* OE lines after 150 mM NaCl treatment. (**b**) MDA contents of WT and *GmERF105* OE lines after 150 mM NaCl treatment. (**c**) SOD activity of WT and *GmERF105* OE lines after 150 mM NaCl treatment. (**d**) CAT activity of WT and *GmERF105* OE lines after 150 mM NaCl treatment. After 16 days of salt treatment, the leaves were selected as samples. The data are mean values ± SDs (* *p* < 0.05, ** *p* < 0.01; Student’s *t*-test). All the experiments included three biological replications.

**Figure 8:**
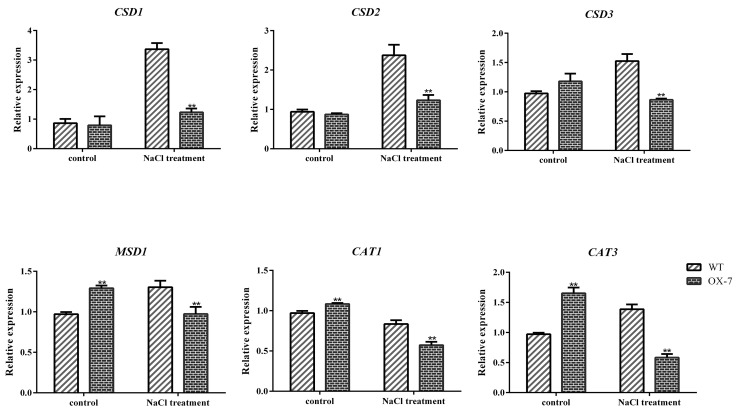
Transcription levels of SOD isoforms and CAT isoforms in WT and *GmERF105* overexpressing plants. The wild-type controls and *GmERF105* transgenic lines plants were grown in pots for two weeks and then irrigated with a solution of 150 mM NaCl for 16 days. Samples were taken from the aboveground part of the Arabidopsis plant. The transcription levels of each gene were analyzed by qRT-PCR using *actin*2 as the reference gene. The data are mean values ± SDs (** *p* < 0.01; Student’s *t*-test). All the experiments included three biological replications.

Previous studies have shown that ROS levels increase dramatically in plants during periods of environmental stress, which can cause serious damage to cell structure. In this study, we measured the content of hydrogen peroxide, which is used to measure ROS levels in plants. Under normal conditions, there was no significant difference between the two groups. After 150 mM NaCl treatment, H_2_O_2_ content was significantly increased generally, and the *GmERF105* OE lines displayed higher accumulation levels than WT (Figure 7a). Furthermore, more MDA was accumulated in overexpressed plants than in WT, with H_2_O_2_ in WT increasing 2.11 times, while that in *GmERF105* OE lines increased 5.63 times (Figure 7b), indicating that the membrane oxidative damage was more pronounced in the overexpressed plants. SOD and CAT enzymes directly eliminate ROS to regulate ROS levels in plant cells. They turn ROS into less active and less toxic hazardous substances. Results demonstrated no significant difference in their enzymatic activity between transgenic and WT lines before the NaCl treatment, but after the treatment, CAT and SOD enzymatic activity were reduced, and it was significantly lower in transgenic plants than in WT plants (Figure 7c,d). In addition, we further detected the transcriptional levels of SOD isoforms (Cu-*ZnSODs*, FeSODs, and MnSOD) and CAT isoforms (CAT1, CAT2, and CAT3). The results showed that the expression levels of CSDs, MSD1, CAT1, and CAT3 in overexpressing *GmERF10*5 plants were lower than those in the wild type under salt stress (Figure 8), while the expression levels of FSDs and CAT2 were not significantly different (Figure S2). In general, these results suggest that the decreased salt tolerance of *GmERF105* transgenic Arabidopsis may be related to the decreased scavenging ability of ROS.

### 2.8. Altered Expression of Stress-Responsive Genes in Transgenic GmERF105 Plants

**Figure 9:**
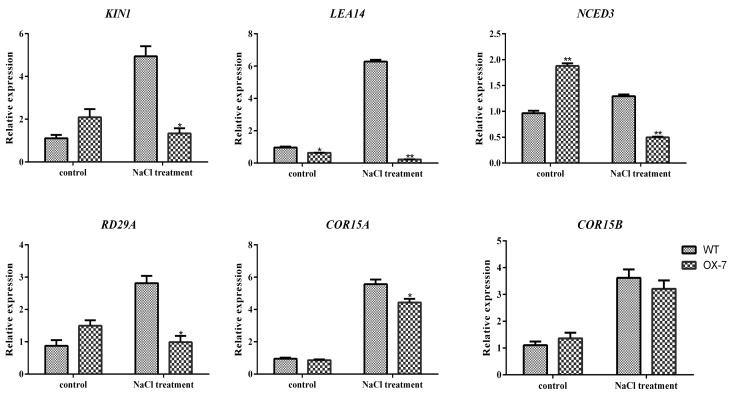
Expression levels of stress-related genes in WT and GmERF105 overexpressing plants. The transcription levels of *KIN1*, *LEA14*, *NCED3*, *RD29A*, *COR15A*, and *COR15B* were analyzed by qRT-PCR using *actin2* as the reference gene. The data are mean values ± SDs (* *p* < 0.05, ** *p* < 0.01; Student’s *t*-test). All the experiments included three biological replications.

To explore the molecular mechanism of *GmERF105* regulation under salt stress, we quantitatively analyzed the expression of six stress-related genes in transgenic *Arabidopsis thaliana* and wild-type plants. The results showed that the expression levels of *KIN1*, *NCED3,* and *RD29A* were higher in *GmERF105*-overexpressed plants under the control condition, while the expression levels of *KIN1*, *NCED3,* and *RD29A* were lower than the wild type under salt stress. The expression level of *LEA14* was inhibited and was lower in *GmERF105* overexpressed plants under both conditions. There was no significant difference between *COR15A* and *COR15B* in wild-type and overexpressed lines under normal growth conditions. However, under salt stress, *COR15A* and *COR15B* expressions were induced and upregulated, and the expression levels in *GmERF105*-overexpressed plants were lower than those in WT plants. In general, the expression levels of *KIN1*, *LEA14*, *NCED3*, *RD29A,* and *COR15A/B* in *GmERF105* overexpressed plants were lower than in the wild type under salt treatment (Figure 9). Therefore, *GmERF105* may negatively regulate the expression of stress-related genes in plants under salt stress.

## 3. Discussion

### 3.1. Transcription Factor GmERF105 Acts as a Negative Regulator in Plant Salt Tolerance Pathways

Biological functional analysis has demonstrated that ERF proteins play a ubiquitous role in plant responses to various abiotic stimuli [27]. ERF proteins have been discovered to play significant roles in response to different abiotic stressors, including but not limited to Arabidopsis [28], rice [29], maize [30], wheat [31], and tobacco [21]. ERF proteins can alter the transcription levels of downstream genes by binding to specific promoter elements, allowing plants to adapt to various environments [32]. In Arabidopsis, *ERF34* plays a crucial role in salt stress response and leaf senescence by directly binding to the *ERD10* and *RD29A* promoter elements [33]. ERF1 regulates specific genes in response to various abiotic stresses by binding to GCC or DRE/CRT elements [34]. Moreover, Wang et al. found that ZmERF21 improves maize drought tolerance by regulating the expression of downstream drought response genes through binding to elements such as GCC-box [26]. In this study, we identified the transcription factor *GmERF105* as a member of the ERF family. Like other ERF family members, GmERF105 is located in the nucleus and can bind to the GCC box. Its expression is induced by salt and drought stress.

Under salt stress, plant growth is inhibited, and a series of physiological and biochemical activities, such as photosynthesis and ion balance, are affected by excessive sodium ion accumulation in cells [35]. Research has identified various genes involved in plant salt stress response, such as the R2R3-MYB family gene *TaODORANT1*, which positively regulates salt tolerance in transgenic tobacco plants by decreasing the accumulation of sodium ions under salt stress [36]. In salt treatment, overexpression of *OsUGE3* enhanced rice salt stress tolerance, and it was found that the sodium ion level in the overexpressed plant was lower [37]. In our study, we found that overexpression of *GmER*F105, a transcription factor containing an AP2 domain, significantly inhibited plant growth and increased sodium ion accumulation in the aboveground part under salt stress. These results suggest that *GmERF105* may decrease plant salt stress tolerance.

### 3.2. GmERF105 Inhibits the ROS Scavenging Systems under Salt Stress

Plants experience high levels of reactive oxygen species (ROS) under salt stress, which can lead to membrane lipid peroxidation and impede plant growth and development [38,39]. However, plants have evolved their own ROS scavenging mechanisms to mitigate cell membrane damage caused by excess ROS. Enzymatic systems, such as SOD and CAT, play crucial roles in scavenging ROS, and an increase in their activity can reduce ROS accumulation and prevent oxidative damage [40]. For instance, SlSTE1 overexpression in plants leads to an increase in antioxidant enzyme (CAT and SOD) activities, reducing ROS accumulation and enhancing salt tolerance [41]. On the other hand, BcWRKY1 transgenic plants exhibit reduced salt tolerance due to the inhibition of ROS signaling and scavenging by lowering APX and SOD activities [42]. In this study, we found that under salt stress, GmERF105-overexpressing plants exhibited significantly lower SOD and CAT activities and higher levels of MDA and H_2_O_2_ compared to the wild type. The regulatory role of LcERF056 in salt tolerance and its effect on ROS-related genes, including lipid transfer proteins, peroxidases, and ribosomal proteins, have been previously reported [43]. Our study further investigated the transcript levels of genes encoding ROS scavenging enzymes and found that the GmERF105 overexpression plants exhibited lower transcript levels of CSD1 and CDS2 compared to the wild type. Consistent with previous research, Cu/Zn-SODs are arguably the most important SODs and their role in plant stress response is supported by their increased expression under stress [44,45,46]. Additionally, we identified GCC-box elements in the promoter regions of CDS1 and CDS2 and confirmed through EMSA experiments that GmERF105 could bind to GCC-box elements in vitro (Figure 3). Therefore, we hypothesize that GmERF105 may have suppressed the transcript levels of ROS scavenging-related genes by binding to GCC-box elements in the promoter regions of ROS scavenging-related genes. Our results suggest that GmERF105 reduces the ability of plants to adapt to salt stress by inhibiting ROS scavenging, ultimately leading to reduced plant viability.

### 3.3. GmERF105 Downregulated the Expression of Stress-Related Genes under Salt Stress

Plants are highly adaptable organisms that possess intricate molecular machinery enabling them to sense and respond to environmental stressors such as drought, high salinity, extreme temperatures, or pathogen attacks. Upon exposure to stress, a wide range of physiological, biochemical, and molecular changes occur within the plant, including the activation of various signaling pathways, the modulation of gene expression patterns, and the accumulation of stress-responsive proteins and metabolites. One critical aspect of plant stress response is the regulation of gene expression. In particular, a subset of genes involved in stress response undergoes significant changes in their expression levels, which enables the plant to cope with and adapt to specific stress conditions [47,48,49,50]. Among them, *KIN1* and *RD29A* are known to be upregulated under various environmental stresses such as low temperature, salt, exogenous ABA, and dehydration [51,52]. *AtLEA14* overexpression in Arabidopsis and yeast resulted in enhanced salinity tolerance, and transcription levels of salt response marker genes *COR15A*, *KIN1*, *RD29B*, and *ERD10* were higher in *AtLEA14* overexpressed plants than in wild-type plants under salt stress [53]. Furthermore, *COR15A/B* has been found to play a critical role in cold and drought stress [54,55]. NCED3 is a key enzyme for ABA biosynthesis, the most important hormone in regulating stress responses [56]. During the salt stress response, ABA acts as a critical secondary signaling molecule, initiating a cascade of responses and regulating gene expression [51]. Our study revealed that *GmERF105* overexpression led to lower transcript levels of stress-responsive genes, including *KIN1*, *LEA14*, *RD29A*, *NCED3*, and *COR15A/B*, in comparison to the wild type under salt stress. These results suggest that *GmERF105* may negatively regulate plant salt tolerance by modulating the expression of stress-responsive genes.

## 4. Materials and Methods

### 4.1. Plant Materials and Growth Conditions

In this study, the soybean cultivar Williams 82 (W82) was utilized. The seeds germinated in vermiculite, and seedlings with uniform growth were selected and cultured in ½-strength Hoagland’s solution. When the first trifoliate leaves are fully unfolded, the seedlings were used for various treatments. The seedlings were moved into a ½-strength Hoagland’s solution with 150 mM NaCl, 50 mM NaHCO_3,_ 100 μM ABA, or 50 μM ACC. All the above cultures were carried out in a culture room with 28 °C/25 °C, 70% relative humidity, and 14 h light/10 h dark period.

Seeds of *Arabidopsis* ecotype Columbia (Col-0) plants were germinated and grown in a growth chamber with the following conditions: 22–24 °C, 60% relative humidity, 100 mol photons m^−2^ s^−1^ light intensity and a 16 h light/8 h darkness photoperiod. The seeds were sown and grown in potting media from germination to harvest. For analysis of gene expression in Arabidopsis, seeds were sown on MS agar plates in darkness for 4 days at 4 °C and then placed in a growth chamber.

### 4.2. Experimental Design

The physicochemical properties and protein structure of GmERF105 were analyzed using bioinformatics methods, while the expression pattern of the GmERF105 gene was analyzed via qRT-PCR. Subsequently, salt tolerance in transgenic *Arabidopsis thaliana* overexpressing GmERF105 was examined through genetic transformation mediated by *Agrobacterium.*

### 4.3. RNA Isolation, cDNA Synthesis, and Quantitative Real-Time PCR

Total RNA from soybean and Arabidopsis was extracted using TRIzol (Sangon Biotech, Shanghai, China), and reverse transcription for the first strand cDNA synthesis was performed with 1 µg of total RNA using a PrimeScript RT Reagent Kit (Vazyme Biotech, Nanjing, China). qRT–PCR analyses were performed using SYBR Premix ExTaq^TM^ II Mix (Vazyme Biotech, Nanjing, China). *Actin3* (GenBank accession No. NP_001276160.2) was used as a reference in soybean. The housekeeping gene *actin2* (GenBank accession No. NP_188508.1) was used as a reference in Arabidopsis. The data were analyzed with the 2^-ΔΔCT^ method, and the primers used for qRT-PCR are listed in Supplementary Table S1.

### 4.4. GmERF105 Gene Isolation and Sequence Analysis

The *GmERF105* gene was isolated from the soybean cultivar W82. The full sequence of *GmERF105* was amplified via PCR with the following primer pair: 5′-TAAAACCATTCACAGTCTTGAACCC-3′ and 5′-CCAACCTAAATCAGAGAGGACAATC-3′. The PCR products were cloned into the pEASY vector (TransGen Biotech, Beijing, China), and the positive clones were sent for sequencing. Multiple sequence alignment analysis was performed using DNAMAN software. Homology analysis of *GmERF105* and the other 13 reference ERF superfamily genes was performed using MEGA 6.0 software through a neighbor-joining method. The amino acid sequences were obtained from GenBank (http://www.ncbi.nlm.nih.gov/genbank/, accessed on 15 January 2023) and Phytozome (http://phytozome.jgi.doe.gov/pz/portal.html/, accessed on 17 January 2023).

### 4.5. Subcellular Localization Analysis

To analyze the subcellular localization of the GmERF105 protein, full-length *GmERF105* without a stop codon was inserted into the *Kpn* I/*Sma* I sites of a pBinGFP4 vector to generate a *GmERF10*5-GFP construct and an empty plasmid was used as a control. The construct was subsequently transformed into *Agrobacterium tumefaciens* strain GV3101, then injected into tobacco with *Agrobacterium tumefaciens* carrying the red fluorescent nuclear localization protein. After 2–3 days, the green and red fluorescence signals in tobacco epidermal cells were observed under a confocal laser-scanning microscope (Olympus FluoView FV1000, Tokyo, Japan). The excitation wavelengths used were 488 nm for GFP and 580 nm for RFP.

### 4.6. Purification of Fusion Proteins and Electrophoretic Mobility Shift Assays

In order to obtain the GmERF105 protein, we inserted the CDS sequence into the PGX-6P-1 vector to create GST-*GmERF105*. Then the recombinant fusion plasmid was transferred into *Escherichia coli* strain BL21 (DE3), and the positive clones were induced with 0.5 mM isopropyl-β-D-thiogalactoside (IPTG) and cultured for 2 h at 160 rpm at 37 °C. For recombinant protein purification, we used a gravity column and GST agarose purification resin according to the manufacturer’s protocols (BBI, Shanghai, China). The probe of GCC-box was 5′-CGCCGCCAATTGCCGCCAATAGCCGCCA-3′(forward) and 5′-GGCGGCTATTGGCGGCAATTGGCGGCG-3′(reverse). The probe of mutant GCC-box (mGCC-box) was 5′-CTCCTCCAATTTCCTCCAATATCCTCC-3′(forward) and 5′-GGAGGATATTGGAGGAAATTGGAGGAG-3′ (reverse). Probes were labeled with 5′6-FAM (fluorescein isothiocyanate) fluorescent dye. Unlabeled probes were used in competition assays. EMSAs were performed using the EMSA Kit (Beyotime, Shanghai, China).

### 4.7. Plant Transformation

Arabidopsis ecotype Col-0 was used for transformation. The 801-bp fragment containing the entire amino acid coding region of *GmERF105* was ligated into the pTF101.1 vector under the control of the CaMV 35S promoter, yielding pTF101.1-*GmERF105*. The construct was subsequently transformed into *Agrobacterium tumefaciens* strain GV3101, and then the target gene was transferred into *Arabidopsis* by the floral-dip method [57].

### 4.8. Phenotypic Analysis of Arabidopsis Tolerance to Salt Stress

To analyze the phenotypes of *GmERF105*-overexpressing (OX) and wild-type (WT) *Arabidopsis* under salt stress, seeds of T_3_ *GmERF105*-overexpressing and WT plants were used. Among them, three transgenic lines with high expression levels were selected. The seed surfaces were sterilized with 10% sodium hypochlorite for 10 min and subsequently washed with deionized water. The sterilized seeds were grown on MS agar plates in darkness for 4 days at 4 °C. Then, the plates were oriented upright and placed in a growth chamber at 22–24 °C, 60% relative humidity, 100 µmol photons m^−2^ s^−1^ light intensity, and 16 h light/8 h darkness photoperiod. For seed germination, seeds were planted on MS solid medium containing 0 mM, 75 mM, 100 mM, and 150 mM concentrations of NaCl. Analysis of salt tolerance in the seedling stage: Arabidopsis seeds were sown in nourishing soil. The 2-week-old Arabidopsis plants were irrigated with a solution of 150 mM NaCl every 4 days for a total of 16 days, and then a camera was used to record the phenotype. Then, fresh weight and sodium ion (Na^+^) concentration were measured. To measure Na^+^ concentration, fresh leaves of WT and overexpressed plants were dried and ground into powder, which was then dissolved in HNO_3_. Ions were determined using an inductively coupled plasma emission spectrometer (Optima 8000, Waltham, MA, USA).

### 4.9. Oxidative Stress Analyses

*GmERF105* overexpression and WT lines were treated with or without salt for 16 days, and the aboveground part of the plant were selected as samples. The content of hydrogen peroxide (H_2_O_2_) and malondialdehyde (MDA) was detected using a kit from Suzhou Grace Biotechnology Company. The activities of two antioxidant enzymes (SOD and CAT) were determined according to the kit instructions (Suzhou Grace Biotechnology Company, Suzhou, China).

### 4.10. Statistical Analysis

All experiments involving each group were performed at least in triplicate. The data are reported as the means ± SD. All the data were analyzed via Student’s *t*-test using GraphPad Prism 6.01 software to assess significant differences between the means.

## 5. Conclusions

**Figure 10:**
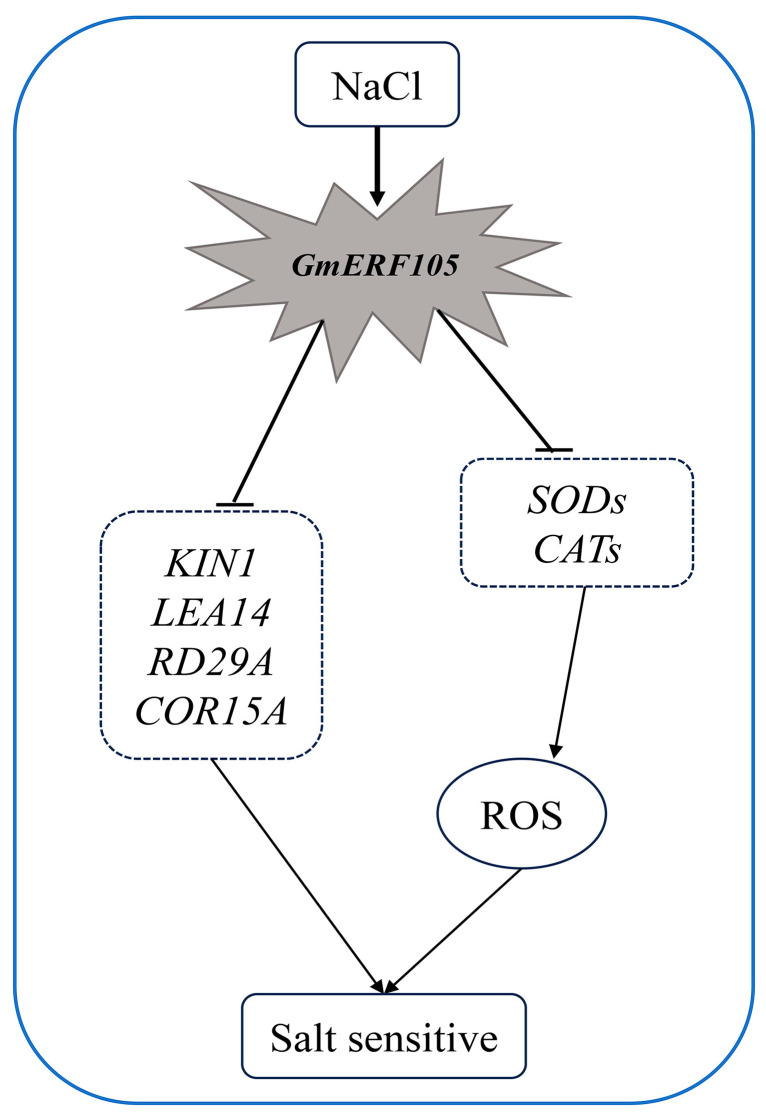
The molecular mechanism model for the regulation of plant salt stress by *GmERF105*. When plants are under salt stress conditions, the transcription level of *GmERF105* increases. *GmERF105* inhibits ROS scavenging capacity and reduces the transcription of stress-related genes in plants, resulting in an increased sensitivity of plants to salt stress.

In this study, we investigated the role of *GmERF105*, a member of the ERF family, in plant adaptation to salt stress. Our results demonstrate that the expression of *GmERF105* is up-regulated by salt, drought, and ABA induction. Furthermore, under salt stress, overexpression of *GmERF105* led to a decrease in plant salt tolerance, as evidenced by reduced seed germination rate and fresh weight and increased sodium content. Additionally, we found that overexpression of *GmERF105* resulted in suppression of SOD and CAT activities, increased ROS levels, and reduced transcription levels of stress-responsive genes in overexpressed *GmERF105* plants. In summary, we have proposed a molecular mechanism model (Figure 10) for the regulation of plant salt stress by *GmERF105*. When plants are exposed to salt stress, the transcription level of *GmERF105* is stimulated. Elevated levels of *GmERF105* can enhance plant sensitivity to salt stress by inhibiting ROS scavenging capacity and reducing the transcription of stress-related genes.

## Data Availability

The datasets used and/or analyzed during the current study are available from the corresponding author upon reasonable request.

## Supplementary Materials

The following supporting information can be downloaded at: https://www.mdpi.com/article/10.3390/plants12163007/s1, Figure S1: qRT-PCR identification of GmERF105 transgenic lines; Figure S2: Transcription levels of FSDs and CAT2 in WT and GmERF105 overexpressing plants; Figure S3: Promoter indicator diagram for CDS1 and CDS2. Table S1: List of the primers in present study.

## Abbreviations

ERF: ethylene response factor; ROS: reactive oxygen species; SOD: superoxide dismutase; CAT: catalase; ABA: abscisic acid; WT: wild type; OX: overexpression.
