# Supplementary material for: Transcription Factor GmERF105 Negatively Regulates Salt Stress Tolerance in Arabidopsis thaliana
Source: Plants (Basel). 2023 Aug 21;12(16):3007. doi: 10.3390/plants12163007 (PMC10459988; doi:10.3390/plants12163007)
Supplement: Supplementary file 1 [file plants-12-03007-s001.zip › Supplementary Figures.pdf]

Transcription Factor *GmERF105* Negatively Regulates Salt Stress Tolerance in *Arabidopsis thaliana*

Lu Li, Zhen Zhu, Juan Liu, Yu Zhang, Yang Lu, Jinming Zhao*, Han Xing* and Na Guo*

[1]National Center for Soybean Improvement, Key Laboratory of Biology and Genetics and Breeding for Soybean, Ministry of Agriculture, National Key Laboratory of Crop Genetics & Germplasm Enhancement and Utilization, College of Agriculture, Nanjing Agricultural University, Nanjing 210095, China

Additional files 1.

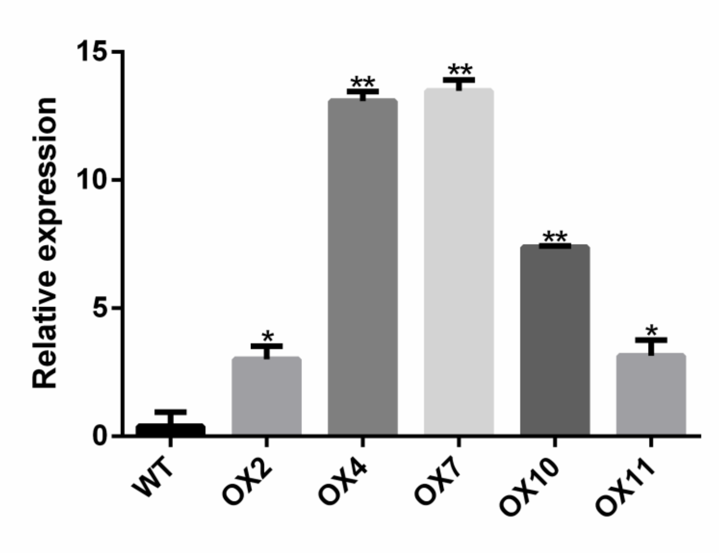

Figure S1. qRT-PCR identification of *GmERF105* transgenic lines.

WT: wild type of Arabidopsis Columbia-0; OX2, OX4, 0X7, OX10, OX11: five transgenic lines of *GmERF105* in T_3 generation. The transcription level of *GmERF105* was analyzed by qRT-PCR using actin2 as the reference gene. Data are means ± SD. Error bars represent the standard error of three replicates.

Additional files 2

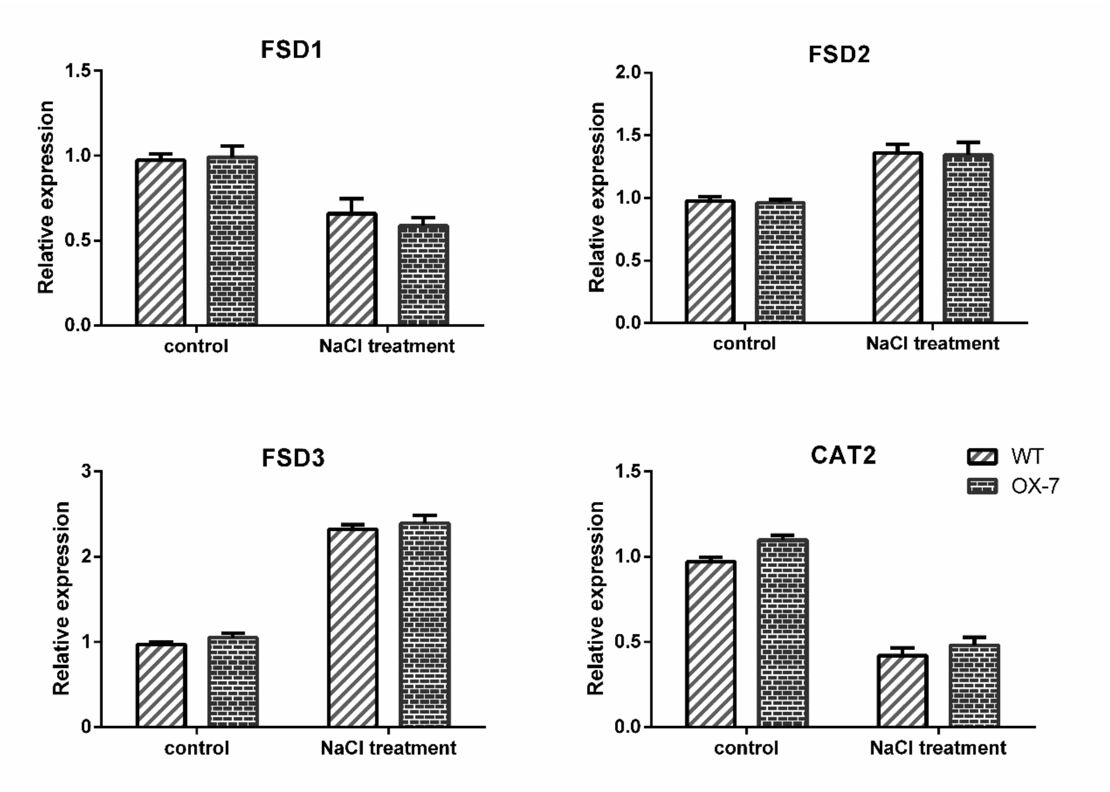

Figure S2. Transcription levels of FSDs and CAT2 in WT and *GmERF105* overexpressing plants

The wild-type controls and *GmERF105* transgenic lines plants were grown in pots for two weeks and then irrigated with a solution of 150 mM NaCl for 16 days. Samples were taken from the aboveground part of the Arabidopsis plant. The transcription levels of each gene were analyzed by qRT-PCR using actin2 as the reference gene. Error bars represent ± SD based on three replicates.

Additional files 3

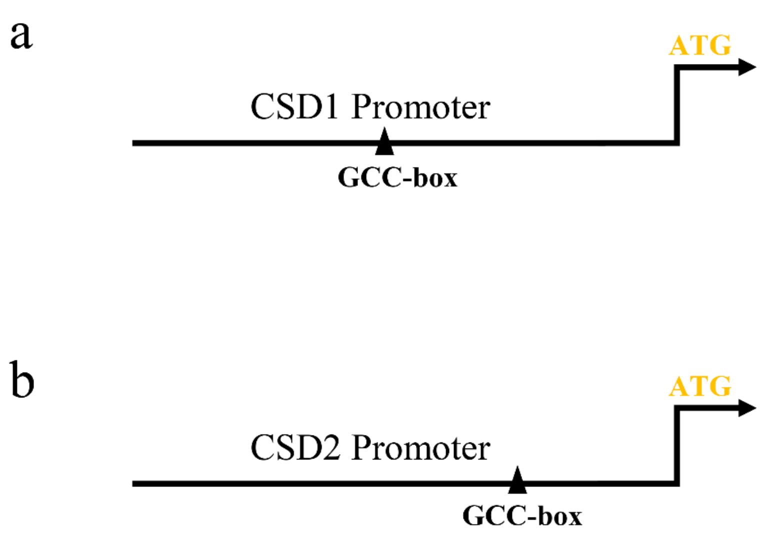

Figure S3. Promoter indicator diagram for *CDS1* and *CDS2*.

GCC-box was indicated by the black triangles.
